# Diversity, distribution of *Puroindoline* genes and their effect on kernel hardness in a diverse panel of Chinese wheat germplasm

**DOI:** 10.1186/s12870-017-1101-8

**Published:** 2017-09-20

**Authors:** Xiaoling Ma, Muhammad Sajjad, Jing Wang, Wenlong Yang, Jiazhu Sun, Xin Li, Aimin Zhang, Dongcheng Liu

**Affiliations:** 1 State Key Laboratory of Plant Cell and Chromosome Engineering, Institute of Genetics and Developmental Biology, Chinese Academy of Sciences, 1 West Beichen Road, Chaoyang District, Beijing, 100101 China; 2 University of Chinese Academy of Sciences, Beijing, 100049 China; 3 Department of Environmental Sciences, COMSATS Institute of Information Technology, Vehari, 61100 Pakistan; 4 The Institute of Forestry and Pomology, Beijing Academy of Agriculture and Forestry Sciences, Beijing, 100093 China

**Keywords:** Common wheat, Kernel hardness, *Puroindoline* genes, EcoTILLING, Allelic variants

## Abstract

**Background:**

Kernel hardness, which has great influence on the end-use properties of common wheat, is mainly controlled by *Puroindoline* genes, *Pina* and *Pinb*. Using EcoTILLING platform, we herein investigated the allelic variations of *Pina* and *Pinb* genes and their association with the Single Kernel Characterization System (SKCS) hardness index in a diverse panel of wheat germplasm.

**Results:**

The kernel hardness varied from 1.4 to 102.7, displaying a wide range of hardness index. In total, six *Pina* and nine *Pinb* alleles resulting in 15 genotypes were detected in 1787 accessions. The most common alleles are the wild type *Pina-D1a* (90.4%) and *Pina-D1b* (7.4%) for *Pina*, and *Pinb-D1b* (43.6%), *Pinb-D1a* (41.1%) and *Pinb-D1p* (12.8%) for *Pinb*. All the genotypes have hard type kernel hardness of SKCS index (>60.0), except the wild types of *Pina* and *Pinb* combination (*Pina-D1a*/*Pinb-D1a*). The most frequent genotypes in Chinese and foreign cultivars was *Pina-D1a*/*Pinb-D1b* (46.3 and 39.0%, respectively) and in Chinese landraces was *Pina-D1a*/*Pinb-D1a* (54.2%). The frequencies of hard type accessions are increasing from 35.5% in the region IV, to 40.6 and 61.4% in the regions III and II, and then to 77.0% in the region I, while those of soft type are accordingly decreasing along with the increase of latitude. Varieties released after 2000 in Beijing, Hebei, Shandong and Henan have higher average kernel hardness index than that released before 2000.

**Conclusion:**

The kernel hardness in a diverse panel of Chinese wheat germplasm revealed an increasing of kernel hardness generally along with the latitude across China. The wild type *Pina-D1a* and *Pinb-D1a*, and one *Pinb* mutant (*Pinb-D1b*) are the most common alleles of six *Pina* and nine *Pinb* alleles, and a new double null genotype (*Pina-D1x*/*Pinb-D1ah*) possessed relatively high SKCS hardness index. More hard type varieties were released in recent years with different prevalence of *Pin-D1* combinations in different regions. This work would benefit the understanding of the selection and molecular processes of kernel hardness across China and different breeding stages, and provide useful information for the improvement of wheat quality in China.

**Electronic supplementary material:**

The online version of this article (10.1186/s12870-017-1101-8) contains supplementary material, which is available to authorized users.

## Background

Wheat (*Triticum aestivum* L.) is one of the most widely grown food crop all over the world with a wide array of food products for human consumption. The kernel hardness is a major determinant of end-use food properties of wheat grain. Kernel hardness refers to the texture of the grain (caryopsis), that is, whether the endosperm is physically hard or soft. This difference in grain texture is due to a 13–15 kDa marker protein, friabilin, which is highly present on the surface of water-washed starch of soft wheat, lower on hard wheat starch and absent on durum wheat starch [1]. The N-terminal sequence analysis of friabilin revealed a mixture of two or more distinct polypeptides [2, 3], which were found to be identical to the two lipid binding proteins, Puroindoline a (PINA) and b (PINB) [4]. The transcripts of *PINA* and *PINB* are controlled by two linked genes, *Pina* and *Pinb*, respectively, located on short arm of chromosome 5D [5]. The presence of wild type *Pina-D1a* and *Pinb-D1a* genes are both necessary to the grain softening of allohexaploid nature of wheat (AABBDD, 2n = 6× =42). However, homologous of the *Pina* and *Pinb* genes are absent on the wheat 5A and 5B chromosomes, and thus durum wheats (*Triticum turgidum* L.) (AABB, 2n = 4× = 28) lacking *Pina* and *Pinb* genes have hard textured grains [6].

Soft grain wheat varieties have wild type (WT) alleles of both *Pina* and *Pinb* genes and any mutation in WT alleles at one or both *Pin* genes gives rise to hard grain texture leading to changed food technological properties [7]. The variation in degree of grain texture hardness is due to a spectrum of alleles and their combinations at *Pina* and *Pinb*, and the number of alleles at *Pina* and *Pinb* detected so far has increased to 23 and 33, respectively [7–10]. Since the basic mechanism by which *Puroindolines* induce soften endosperm is not well known, genotype-phenotype associations are useful to estimate the kernel hardness effect of a *Pin* allele. The absence or altered primary structure of one of PINA and PINB will result in a hard grain texture, and among commercial wheat cultivars the most prevalent hard genotypes are the absence of PINA or the altered primary structure of PINB with null alleles of *Pina* and *Pina-D1a*/*Pinb-D1b*, respectively [11–13]. However, cultivars with null alleles of *Pina* have been proposed harder than those with *Pina-D1a*/*Pinb-D1b* [13–19]. Moreover, the genotype with *Pina-D1b* (the null allele of *Pina*) may have poor milling quality and relatively inferior processing quality for steamed bread, pan bread, and Chinese noodles [16]. This null allele has been molecular characterized with a large deletion of 15,380 bp through a primer walking strategy and a diagnostic sequence tagged site (STS) marker has also been developed spanning the deletion fragment [20]. Moreover, on chromosomes 7A, 7B and 7D in bread wheat, homologous *Pinb* genes have been found with more than 70.0% similarity, though their function is elusive [21–23]. Although most of the known hardness alleles confer large and somewhat similar changes in endosperm texture relative to soft wheat, the discovery of new alleles could broaden the genetic background for kernel hardness and provide industry with grains more suitable for a variety of end-uses. Furthermore, different combinations of *Pina* and *Pinb* alleles in common wheat determine the grain textural classes with diverse end-use characteristics [24]. Thus, knowledge on the *Puroindoline* allelic composition in a diverse panel of germplasm is prerequisite for the parental selection for developing varieties with desired kernel hardness.

The research herein presents the analysis of *Puroindoline* allelic variations, genotypes and their association with kernel hardness in a diverse panel of wheat accessions comprising 1539 Chinese cultivars, 107 Chinese landraces and 141 foreign accessions. A subset of 623 accessions was evaluated for two consecutive years to assess environmental effect on SKCS index of kernel hardness. Accessions collected in Beijing, Hebei, Shandong and Henan were analyzed in detail to reveal the hardness trends along with breeding stages and the prevalence of *Pina* and *Pinb* alleles. The knowledge generated in this study about the allelic effect on and dynamics of kernel hardness, and the allele distribution across China would enhance breeder’s choice for suitable parent selection to develop cultivars of desired kernel hardness, and improve the understanding on the patterns of kernel hardness across different wheat zones in China. The new double null mutants would provide an alternative option for kernel hardness improvement.

## Results

### High phenotypic variation in kernel hardness

To assess the kernel hardness in Chinese wheat germplasm, 1646 accessions were collected from nine wheat cultivation regions in China and grown in Beijing in 2009–2010 cropping season, along with 141 foreign accessions from USA, Australia, Europe and Japan (Table 1). Of the Chinese accessions, 1582 accessions were from cultivation region I (Northern winter wheat region), II (Yellow and Huai River Valley winter wheat region), III (Middle and Low Yangtze Valley winter wheat region) and IV (Southwestern winter wheat region), and the remaining 64 accessions were from other spring wheat cultivation regions since most spring wheat from these regions hardly survive through the cold winter season in Beijing. The kernel hardness of harvested samples in 2009–2010 growing season measured with SKCS showed that 1787 accessions contained a wide range of SKCS index varying from 1.4 to 102.7 (Table 2). The major class was of hard type including 1075 accessions (60.2%), which were from most wheat cultivation regions of China, except Qing-Tibetan Plateau spring-winter wheat region (cultivation region IX) (Table 3). The SKCS index of hard type ranged from 60.1 to 102.7 with mean value of 72.4, and about 93.0% accessions have a hardness index lower than 85.0. The soft type class consisted of 523 accessions (29.2%) with SKCS index ranging from 1.4 to 40.0 and mean value of 25.1, while the medium type class with medium kernel hardness included only 189 accessions (10.6%), ranging from 40.0 to 60.0 with mean value of 54.1 (Table 2).

**Table 1:** Number and types of accessions for kernel hardness measurement and *Puroindoline-D1* detection

	Region^a^	Cultivars	Landraces	Total
China	I	244	4	248
	II	1065	25	1090
	III	81	25	106
	IV	95	43	138
	V	2	1	3
	VI	9	3	12
	VIII	32	4	36
	IX	1	2	3
	X	10	0	10
Foreign				141
Total		1539	107	1787

^a^Definition of wheat cultivation regions: I (Northern winter wheat region), II (Yellow and Huai River Valley winter wheat region), III (Middle and Low Yangtze Valley winter wheat region), IV (Southwestern winter wheat region), V (Southern winter wheat region), VI (Northeastern spring wheat region), VIII (Northwestern spring wheat region), IX (Qing-Tibetan Plateau spring-winter wheat region) and X (Xinjiang winter-spring wheat region)

**Table 2:** Distribution of kernel hardness in and percentage of soft, hard and medium wheats

Type	Number	Freq. (%)	SKCS index	
			Mean	Range
Soft	523	29.2	25.1	1.4–40.0
Hard	1075	60.2	72.4	60.1–102.7
Medium	189	10.6	54.1	40.0–60.0

**Table 3:** Region-wise percent distribution of soft, hard and medium wheats in two different origins and nine wheat cultivation regions in China^a^

	China (1646)									Foreign (141)	Freq. (%)
	I	II	III	IV	V	VI	VIII	IX	X		
Soft	10.5	27.6	51.9	55.8	66.7	66.7	30.6	100.0	30.0	25.5	29.2 (522)
Hard	77.0	61.4	40.6	35.5	33.3	33.3	63.9	0.0	60.0	63.1	60.2 (1075)
Medium	12.5	11.0	7.5	8.7	0.0	0.0	5.6	0.0	10.0	11.3	10.6 (190)

^a^The values in parentheses are the number of accessions and the wheat cultivation regions are defined in Table 1

To determine the environment effect on kernel hardness, 623 accessions were grown on the same station during 2009–2010 and 2010–2011 growing seasons using the same agronomic practices and phenotyped for SKCS. The ranges of SKCS index for the year 2009–2010 were of 1.4 to 97.8 with an average of 49.2 and these for 2010–2011 were 1.1 to 98.0 with an average of 48.0. For the environment effect, the analysis of variance (ANOVA) showed non-significant with *P* value of 0.39 (Additional file 1: Table S1) between 2 years’ data, and thus we considered the SKCS data of 2009–2010 valid for association analysis between SKCS index of kernel hardness and genotypes of *Pina* and *Pinb*.

### Classified regional distribution of hard and soft kernel accessions

Based on the kernel hardness, accessions from nine wheat growing regions in China were analyzed, region I (Northern winter wheat region) had the highest number of hard textured type wheat accessions, 77.0%, and the regions II (Yellow and Huai River Valley winter wheat region), VIII (Northwestern spring wheat region), X (Xinjiang winter-spring wheat region) and foreign accessions were also dominantly represented by hard type wheat, around 60.0% (Table 3). Conversely, the regions III (Middle and Lower Yangtze Valley winter wheat region), IV (Southwestern winter wheat region), V (Southern winter wheat region) and VI (Northeastern spring wheat region) were represented by soft type wheat with about 50.0% prevalence (Table 3). All the regions and foreign accessions were represented by all three kernel hardness types, except regions V and VI that did not contain medium type accessions and IX did not have hard and medium types. This might be due to only a few available accessions collected from these regions (Table 3).

In China, the winter wheat growing regions IV (Southwestern winter wheat region), III (Middle and Low Yangtze Valley winter wheat region), II (Yellow and Huai River Valley winter wheat region) and I (Northern winter wheat region) extends from South to North along with the increase of latitude. Regarding to hardness type in different wheat regions, we found that the frequencies of hard type accessions are increasing from 35.5% in the region IV, to 40.6 and 61.4% in the regions III and II, and then to 77.0% in the region I, while those of soft type are accordingly decreasing along with the increase of latitude (Table 3). This phenomenon was also observed in landraces (Additional file 1: Table S2). More soft accessions present in the regions III and IV than that in the region II, and no soft accessions were detected in the region I (Additional file 1: Table S2).

### Allelic variants of *Pina* and *Pinb* genes

**Fig. 1:**
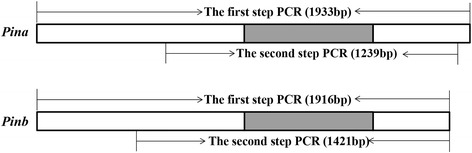
Diagrams of PCR amplification for *Pina* and *Pinb*. The gray boxes show coding regions for *Pina* and *Pinb*, respectively. The first step PCR reaction of *Pina* and *Pinb* (Pina-OutF/R, Pinb-OutF/R) used for amplifying *Pina* (1933 bp) and *Pinb* (1916 bp) genomic sequence. The second step PCR reaction of *Pina* and *Pinb* (Pina-InF/R, Pinb-InF/R) used for amplifying *Pina* (1239 bp) and *Pinb* (1421 bp) genomic sequence

The nested PCR and modified EcoTILLING analysis was performed with allele specific primers to detect allelic variants of *Pina* and *Pinb* genes (Fig. 1; Additional file 1: Table S3). Of the analyzed 1787 accessions, six *Pina* allelic variants including the wild type (*Pina-D1a*) and five mutants were characterized (Table 4). Except *Pina-D1l* has a cytosine deletion as comparing to *Pina-D1a* (Additional file 4: Figure S3A), the remaining mutants belong to different types of *Pina* null mutant (Table 4). The wild type *Pina-D1a* was the most common allele observed in 1616 accessions (90.4%) followed by *Pina-D1b* in 133 accessions (7.4%), while the remaining variants, *Pina-D1l*, *Pina-D1r*, *Pina-D1s* and *Pina-D1x* were rare alleles, present in a few samples (Table 4). Nine allelic variants were identified at *Pinb*, of which *Pinb-D1b*, *Pinb-D1c*, *Pinb-D1d*, *Pinb-D1p*, *Pinb-D1q* and *Pinb-D1u* have SNP mutations, and *Pinb-D1ah* and *Pinb-D1ai* belong to different types of *Pinb* null mutant (Table 4; Additional file 4: Figure S3B). Only three variants *Pinb-D1b*, *Pinb-D1a* and *Pinb-D1p* (43.6, 41.1 and 12.8%, respectively) were common in these allelic variants (Table 4). For the null alleles of *Pina* and *Pinb*, the *Pina-D1x* as a novel null variant was different from previously reported alleles with various fragment deletions (*Pina-D1b*, *Pina-D1r* and *Pina-D1s*) and detected in seven Chinese cultivars and three foreign accessions. There are two novel *Pinb-null* alleles, one is *Pinb* absent but *Pina* present, so this *Pinb-null* allele was designated as *Pinb-D1ai*, and detected only in two accessions; the other *Pinb-null* allele was named as *Pinb-D1ah* and observed in the ten accessions containing the *Pina-D1x* allele, in which the simultaneous deletion of the large segment containing *Pina* and *Pinb* genes might have occurred.

**Table 4:** Distribution of *Pina* and *Pinb* alleles in total 1787 accessions

	*Pina* ^a^						*Pinb* ^a^								
	*D1a*	*D1b*	*D1l*	*D1r*	*D1s*	*D1x*	*D1a*	*D1b*	*D1c*	*D1d*	*D1p*	*D1q*	*D1u*	*D1ah*	*D1ai*
Cultivars	1414	113	1	2	2	7	589	721	0	13	201	7	0	7	1
Landraces	86	4	3	8	6	0	78	4	0	1	18	2	3	0	1
Foreign	116	16	0	1	5	3	68	55	2	4	9	0	0	3	0
Total	1616	133	4	11	13	10	735	780	2	18	228	9	3	10	2

^a^The values are the number of accessions for each allele

Regarding the distribution of allelic variants across Chinese cultivars, landraces and foreign accessions, frequency of each allelic variant of *Pina* and *Pinb* genes was calculated. For *Pina* gene, the wild type allele *Pina-D1a* was predominant in Chinese cultivars, landraces and foreign accessions with frequencies of 91.9, 80.4 and 82.3% in the respective groups. Among the mutants, the most frequent allele was *Pina-D1b* in Chinese cultivars and foreign accessions with frequency of 7.3 and 11.3%, respectively, whereas *Pina-D1r* (7.5%) was the most in landraces, followed by *Pina-D1s* (5.6%) and *Pina-D1b* (3.7%). The variants *Pina-D1l* and *Pina-D1x* were missing in foreign and landrace accessions, respectively (Table 4). For *Pinb* gene, the highest frequency of the wild type allele *Pinb-D1a* was present in Chinese landraces (72.9%), followed by foreign accessions (48.2%) and Chinese cultivars (38.3%), which has an opposite pattern to that of *Pina-D1a*. Among the mutants, *Pinb-D1b* and *Pinb-D1p* had frequencies of 46.8 and 13.1% in Chinese cultivars, respectively, and other four variants were rarely present (totally less than 2.0%); In foreign accessions, *Pinb-D1b* and *Pinb-D1p* were also prevalent with frequencies of 39.0 and 6.4%, but other three variants (*Pinb-D1c*, *Pinb-D1d* and *Pinb-D1ah*) had relatively high frequencies as compared with those in Chinese accessions, whereas *Pinb-D1b* was present in only 3.7% Chinese landraces, and *Pinb-D1p* was the most present mutant (16.8%) in Chinese landraces (Table 4). Interestingly, the allele *Pinb-D1c* was missing in Chinese cultivars and landraces but was found in foreign accessions, while the allele *Pinb-D1u* was completely absent in Chinese cultivars and foreign accessions and was only detected in Chinese landraces, Zhushimai, Zhugoumai and Dajinbaihuazi (Table 4). Those three landraces are found in Yunnan and Sichuan provinces, belonging to Southwestern winter wheat region (IV), and they could be exploited to incorporate this novel allele into Chinese wheat cultivars. Moreover, the variants *Pina-D1l*, *Pinb-D1q* and *Pinb-D1ai* were missing in foreign accessions, and the double mutant *Pina-D1x*/*Pinb-D1ah* was not detected in Chinese landraces. The different allelic frequencies in three type accessions suggested that the *Pina* and *Pinb* were artificially selected by breeders.

To assess the diversity at *Pina* and *Pinb* genes, Nei’s diversity index (*H*) was calculated in the total collection of 1787 accessions. The *H* value at *Pina* and *Pinb* were 0.18 and 0.62, respectively. At *Pina*, the highest diversity (0.34) was observed in 107 Chinese landraces, followed by 0.31 in the group of 141 foreign accessions, and the least (0.15) was observed in 1539 Chinese cultivars. At *Pinb*, Chinese cultivars and foreign accessions had similar high diversity (0.62), and Chinese landraces possessed a lower diversity (0.43), which was different from that of *Pina*. These data demonstrated that more allelic variants of *Pinb* with higher frequencies were retained in breeding process than those of *Pina*.

### Association analysis between genotype and kernel hardness

To investigate the effect of *Pina* and *Pinb* on kernel hardness, genotypes were assayed on six *Pina* and nine *Pinb* variants. Of the 15 *Pina* and *Pinb* combinations (Table 5), eight were the *Pina* wild type (*Pina-D1a*) with eight *Pinb* variants, occupying a 90.4% accessions, three were *Pina-D1b* and three *Pinb* variants with a total frequency of 7.4%, and the remaining four combinations were formed individually by four *Pina* variants with corresponding *Pina* variants. At *Pinb* genotypes, five were formed by the wild type (*Pinb-D1a*) with five *Pina* variants, and two were *Pinb-D1b* and two *Pina* variants, in which *Pinb-D1a* and *Pinb-D1b* genotypes occurred in 41.1 and 43.6% accessions, respectively (Table 5). The most abundant combination was *Pina-D1a*/*Pinb-D1b* (43.1%), followed by *Pina-D1a*/*Pinb-D1a* (32.8%), *Pina-D1a*/*Pinb-D1p* (12.5%) and *Pina-D1b*/*Pinb-D1a* (6.7%) with corresponding average kernel hardness of 67.7, 31.0, 68.0 and 79.3. The other 11 combinations were rare (≤1.0%) with a total frequency of 4.8%, however these combinations had higher average kernel hardness (≥60.0), of which that of *Pina-D1x*/*Pinb-D1ah* was 91.1, at least 10 hardness index higher than all the remaining genotypes (Table 5).

**Table 5:** Association of *Puroindoline-D1* alleles with SKCS index and their distributions in Chinese cultivars, Landraces and foreign accessions

Genotype	SKCS index		Cultivars	Landraces	Foreign
	Mean	Range			
*Pina-D1a/Pinb-D1a*	31.0	1.4–92.5	483	58	46
*Pina-D1a/Pinb-D1b*	67.7	11.0–102.7	712	4	55
*Pina-D1a/Pinb-D1c*	60.0	45.1–74.9	0	0	2
*Pina-D1a/Pinb-D1d*	63.6	48.5–73.6	13	1	4
*Pina-D1a/Pinb-D1p*	68.0	16.1–88.6	198	17	9
*Pina-D1a/Pinb-D1q*	66.2	57.0–78.3	7	2	0
*Pina-D1a/Pinb-D1u*	65.9	58.4–78.4	0	3	0
*Pina-D1a/Pinb-D1ai*	78.6	65.6–91.6	1	1	0
*Pina-D1b/Pinb-D1a*	79.3	19.7–95.6	101	3	16
*Pina-D1b/Pinb-D1b*	68.9	19.9–75.5	9	0	0
*Pina-D1b/Pinb-D1p*	71.1	66.3–82.4	3	1	0
*Pina-D1l/Pinb-D1a*	74.3	68.8–84.2	1	3	0
*Pina-D1r/Pinb-D1a*	76.7	59.9–94.1	2	8	1
*Pina-D1s/Pinb-D1a*	67.1	53.4–86.2	2	6	5
*Pina-D1x/Pinb-D1ah*	91.1	77.1–97.8	7	0	3
Total			1539	107	141

Regarding to the distribution of 15 combinations of *Pina*/*Pinb* alleles in three groups, 13, 12 and 9 were observed in Chinese cultivars, Chinese landraces and foreign accessions, respectively (Table 5). In Chinese cultivars, *Pina-D1a*/*Pinb-D1b* (46.3%), *Pina-D1a*/*Pinb-D1a* (31.4%), *Pina-D1a*/*Pinb-D1p* (12.9%) and *Pina-D1b*/*Pinb-D1a* (6.6%) were predominant, and those predominant combinations also had high frequencies in foreign accessions, while *Pina-D1r*/*Pinb-D1a* (7.5%) and *Pina-D1s*/*Pinb-D1a* (5.6%) were predominant in Chinese landraces, along with *Pina-D1a*/*Pinb-D1a* (54.2%) and *Pina-D1a*/*Pinb-D1p* (15.9%) (Table 5). In addition, some rare combinations were observed in some specific samples, e.g. *Pina-D1a*/*Pinb-D1u* (2.8%) in Chinese landraces, *Pina-D1a*/*Pinb-D1c* (1.4%) in foreign accessions, and *Pina-D1b*/*Pinb-D1b* (0.6%) in Chinese cultivars. All in together, the Nei’s diversity, allelic and genotype frequency results show the promising use of Chinese landraces and foreign accessions to broaden the genetic base of modern Chinese cultivars, and the frequency difference of combinations in three groups suggested that the *Pina* and *Pinb* genes might have been broadly selected during the breeding process (Table 5).

### Selection of kernel hardness and allele preference during breeding process

To investigate the hardness trend and allelic variant preference during Chinese wheat breeding process, 1108 cultivars bred in Beijing, Hebei, Shandong and Henan with clear released time were selected (Table 6). In each province, based on the released year, cultivars were separated into two groups, before year 2000 and after 2000 (including 2000), for the kernel hardness was considered as one of important criterions for new hard wheat varieties released in north China in year 2000 and later. The average kernel hardness was calculated in each group, along with numbers and hardness of three kernel categories (hard, medium and soft) (Table 6). Varieties released after 2000 in Beijing, Hebei, Shandong and Henan had an average kernel hardness index of 69.2, 68.3, 63.7 and 61.1, respectively, significantly higher than that released before 2000 in all four provinces, which demonstrated that the kernel hardness has been greatly improved in the new century. The increase of hardness index after 2000 might be due to the higher frequency of hard type varieties, e.g. 90.4 Vs 65.1% in Beijing, 86.5 Vs 42.2% in Hebei,73.2 Vs 48.7% in Shandong, and 67.3 Vs 30.7% in Henan. Interestingly, the frequencies of hard type varieties released after 2000 were increasing along with the latitude, resulting in an increase of the average hardness index from Henan (generally lower latitude) to Beijing (higher latitude). Moreover, slight differences of the average hardness index were observed in hard type varieties in different groups and provinces, and this could be attributed mainly to the frequencies of combinations of *Pina* and *Pinb* alleles since they preserved different effects on kernel hardness (Table 5). In the hard type varieties, *Pina-D1a*/*Pinb-D1b*, *Pina-D1a*/*Pinb-D1p* and *Pina-D1b*/*Pinb-D1a* were three dominant genotypes, occupying 90.0% or more varieties in each groups. However, these genotypes were differentially selected by breeders in different stages and provinces. For example, *Pina-D1a*/*Pinb-D1p* was a dominant genotype with a percentage of 44.0% in the group of varieties released after 2000 in Beijing, while the preference of *Pina-D1a*/*Pinb-D1b* was observed before 2000; the frequency of *Pina-D1b*/*Pinb-D1a* has been greatly improved in the group after 2000 in Shandong; *Pina-D1b*/*Pinb-D1a* was seldom detected in varieties from Hebei and released before 2000 in Shandong. These data demonstrated that combinations of *Pina* and *Pinb* genes have been widely selected in different breeding stages in different regions.

**Table 6:** Frequencies of hard, medium and soft wheats bred in different periods in Beijing, Hebei, Shandong and Henan, and their prevalent genotypes in hard wheat

Location	Period^a^ (year 2000)	Number	SKCS index		Freq. (%)			Aver. Hardness Freq. (%)			Genotype freq. in hard wheat		
			Mean	Range	Hard	Medium	Soft	Hard	Medium	Soft	*Pina-D1a*/ *Pinb-D1b*	*Pina-D1a*/ *Pinb-D1p*	*Pina-D1b*/ *Pinb-D1a*
Beijing	after	83	69.2	10.0–85.5	90.4	4.8	4.8	72.5	57.1	20.7	37.3	44.0	10.7
	before	106	58.8	12.1–92.5	65.1	16.0	18.9	69.7	52.6	26.7	59.4	20.3	14.5
Hebei	after	281	68.3	13.0–94.2	86.5	8.2	5.3	72.0	56.1	26.6	64.2	16.9	9.9
	before	128	48.4	11.0–90.4	42.2	18.8	39.1	68.4	53.0	24.5	61.1	29.6	1.9
Shandong	after	108	63.7	1.4–95.6	73.2	6.5	20.4	76.5	55.0	20.5	55.7	13.9	20.3
	before	156	50.4	11.1–92.7	48.7	10.3	41.0	71.6	52.9	24.7	75.0	17.1	1.3
Henan	after	107	61.1	6.7–95.0	67.3	13.1	19.6	72.8	53.2	26.3	75.0	8.3	12.5
	before	140	42.2	1.5–83.4	30.7	13.6	55.7	71.1	52.5	23.7	67.4	11.6	16.3

^a^after year 2000 means accessions or varieties bred and released in 2000 or later, and before year 2000 means released in 1999 or before

### The novel alleles at *Pina* and *Pinb*

Several new types of alleles of *Pina* and *Pinb* were also detected in this study. According to the catalogue of gene symbols [6, 9], a synonymous allele ‘C 321 T’ of *Pina-D1a* was designated as *Pina-D1y* (Additional file 2: Figure S3), and its kernel hardness index was 20.5 ± 12.7, categorized to the soft wheat class, which indicated that the synonymous allele of *Pina-D1a* had non-significant effect on kernel hardness. The null alleles at both *Pina* and *Pinb* loci (*Pina-D1x*/*Pinb-D1ah*) were observed in ten accessions (0.6%), of which three were detected in foreign accessions and seven were from Chinese cultivars, including five from Yellow and Huai River Valley winter wheat region (II), and one each from region III and VI (Table 5; Additional file 2: Figure S1; Additional file 3: Figure S2). The SKCS index of these double null mutants ranged from 77.1 to 97.8 with mean of 91.1, which had highest average kernel hardness among 15 combinations of *Pina* and *Pinb* genes and were comparable with that of durum wheat (88.1 ± 15.4) (Additional file 1: Table S4; Table 5).

In effort to further illustrate the deletion size and position in these double null mutants, nine primer pairs (Additional file 1: Table S3) specific to chromosome 5D were designed surrounding the *Pina* and *Pinb* genes with the availability of the BAC sequence (CT009735) in NCBI. Based on the primer walking strategy, amplicons with expected fragment size could be observed with primer sets Pina-1, Pina-7, Pina-8 and Pina-9, whereas no targeted fragment was amplified with primer sets through Pina-2 to Pina-6 in four Chinese cultivars Yunfengzao 21, 06–01216, Kelao 4 and Shan 150 and one foreign accession NIL-Novos 67 (Additional file 1: Table S4; Additional file 2: Figure S1). These absent amplicons revealed a 25-kb deletion at least from −435 to +24,592 bp (reference to ATG of *Pina*) on these five accessions compared with the BAC sequence of Chinese Spring (CT009735) containing the *Pina* and *Pinb* genes. However, no expected fragments were detected with all the primer sets through Pina-1 to Pina-9 in three Chinese accessions Hedong TX-008, Xinong 8925–13, and 91G149/Chang 128,865, and one foreign accession Vendvr, which provided a large fragment deletion in these accessions (at least 90-kb, from −21,803 to +68,481 bp referring to ATG of *Pina* in Chinese Spring BAC sequence CT009735 in NCBI). Moreover, expected fragments of amplicons were only gained with primer sets Pina-7, Pina-8 and Pina-9, but not through Pina-1 to Pina-6 in foreign accession Victory, demonstrating an at least 63-kb deletion from −21,803 to 41,844 bp of Chinese Spring BAC sequence (CT009735) on chromosome 5DS (Additional file 1: Table S4). All these data with primer walking strategy suggested that the *Pina* and *Pinb* gene regions were completely deleted in these double null mutants, which confirmed the EcoTILLING data and the durum-like kernel hardness of these accessions, though further analysis is needed to elucidate the exact deletion sizes. However, these double null mutants with extremely high kernel hardness provided an elite germplasm resource for kernel hardness improvement in wheat breeding though molecular marker assisted selection.

### Double null mutants have durum-like starch granules and protein matrix

**Fig. 2:**
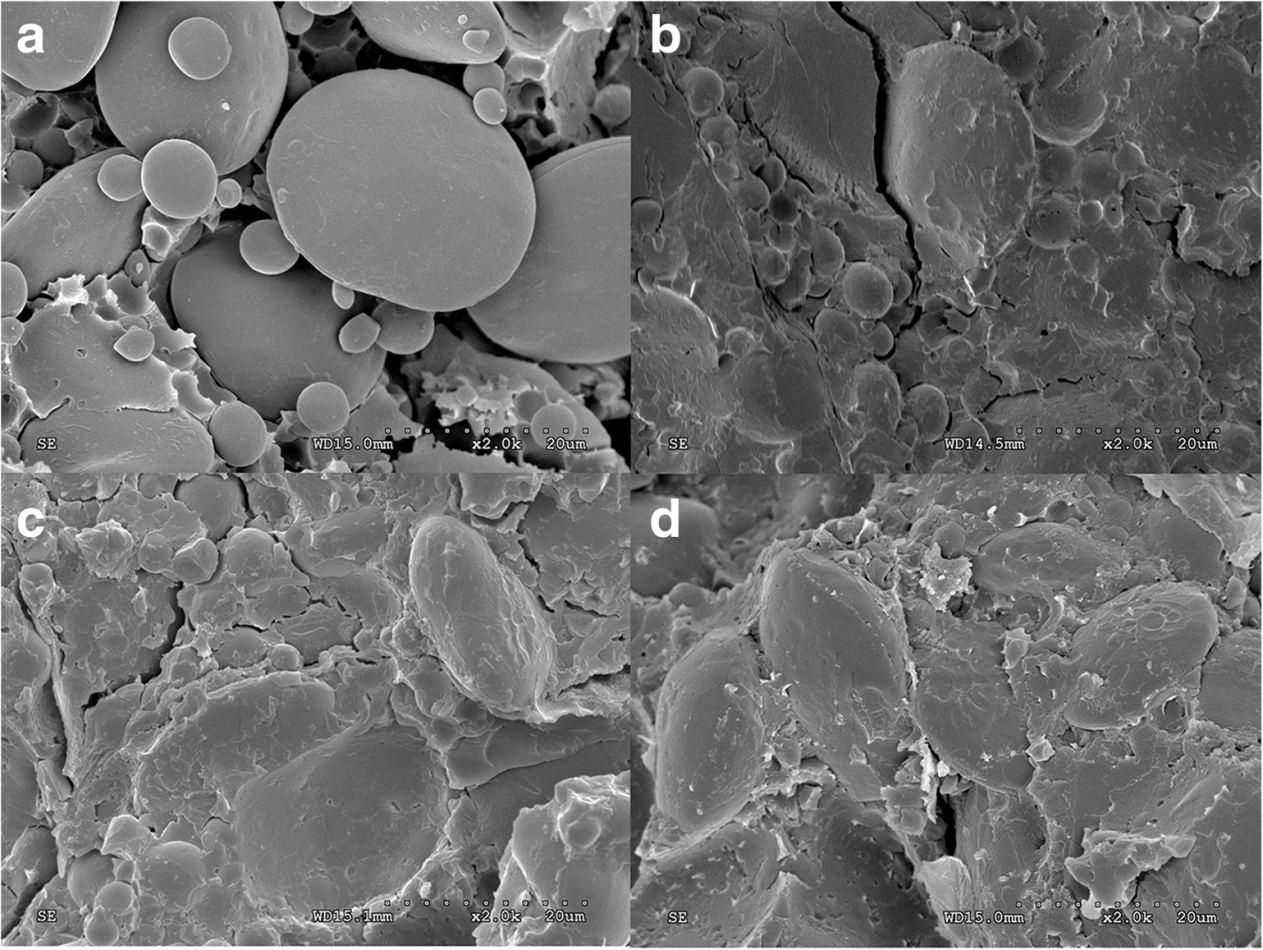
Scanning electron micrographs of the central endosperm region of double null mutants of *Pina* and *Pinb* genes. **a** Freeze-fractured grain of wild type accession (Chinese Spring, soft type control). **b** Durum wheat accession (Neixiang 4184; *Pin null*: both genes deleted, very hard control). **c** Xinong 8925–13 (*Pina-D1x/Pinb-D1ah*). **d** Yunfengzao 21 (*Pina-D1x/Pinb-D1ah*)

SEM has been considered as a straightforward method for determining the adhesion between protein matrix and starch granules [25, 26]. In this study, SEM was performed with one wild type accession (Chinese Spring), two double null accessions (Xinong-8925-13 and Yunfengzao 21) and one durum wheat accession (Neixiang 4184). The SEM images revealed large, medium and small round to spherical starch granules in all four accessions (Fig. 2a-d), and the basic differences were of the adhesion between starch granules and protein matrix among the wild type (*Pina-D1a*/*Pinb-D1a*) (Fig. 2a) and the double null mutants (*Pina-D1x*/*Pinb-D1ah*) (Fig. 2c, d). Starch granules and protein matrix are clearly separated without significant adhesions in-between in the wild type soft textured Chinese Spring, while starch granules were strongly adhered to protein matrix in durum wheat Neixiang 4184 (Fig. 2a, b). More medium and small starch granules were observed in durum wheat, which was prone to be adhered to protein matrix, resulting in higher kernel hardness. SEM images of the double null mutants Xinong 8925-13 (*Pina-D1x*/*Pinb-D1ah*) and Yunfengzao 21 (*Pina-D1x*/*Pinb-D1ah*) revealed that both double null mutants had strong adhesion between protein matrix and starch granules, narrow space between starch granules, and more medium and small starch granules, which were similar to that of durum wheat but completely different from that of Chinese Spring. This phenomena and extreme high kernel hardness confirmed the deletion of both *Pina* and *Pinb* genes in Xinong 8925-13 and Yunfengzao 21 for durum wheat lacking both *Pina* and *Pinb* genes used to have high kernel hardness. Moreover, the protein matrix appeared differently between hexaploid and durum wheat, which has more flake-shape fragments on the surface of starch granules in Chinese Spring and both mutants (Fig. 2a-d). The SKCS and SEM analysis conclusively demonstrated that high kernel hardness was attributed to durum-like grain structure of double null mutants, and both *Pina* and *Pinb* genes might play a vital role in kernel hardness development.

## Discussion

Due to their important roles in determining kernel hardness, the allelic variation of *Pina* and *Pinb* genes have been widely investigated using wheat germplasms from different countries and regions [5, 9–11, 13, 24, 27]. The methods used for characterizing *Pina* and *Pinb* alleles were improved from SDS-PAGE analysis, cloning and sequencing of the alleles in a few lines to EcoTILLING [5]. The optimized EcoTILLING approach was exploited to investigate *Pina* and *Pinb* alleles in the 225 micro-core collection (MCC) accessions of Chinese wheat germplasm [10]. Compared to previous techniques, EcoTILLING approach is highly efficient on time and cost for largely reducing the repetitive work of sequencing the most frequent *Pina-D1a*, *Pinb-D1a* and *Pinb-D1b* alleles. On the other hand, the detection of novel alleles was directly based on the nucleotide sequences, which improved the discovery of novel variants with electrophoretic mobility similar to previously known alleles in SDS-PAGE analysis. The differentiation of *Pinb-D1x* allele (14.5 kDa) from *Pinb-D1ab* (14.4 kDa) was due to the distinct advantage of EcoTILLING approach [10] over SDS-PAGE analysis that might had regarded the two alleles as identical. So the high throughput EcoTILLING approach was exploited in our research with minor modifications. On the one hand, gene cloning method was applied to get the plasmid DNA containing wild type genotype (*Pina-D1a*/*Pinb-D1a*) which minimized the use of control DNA templates and made the adjustment of PCR products concentration more effective than the wild type genomic DNA. On the other hand, nested PCR was used to improve the sensitivity and specificity.

The wide range of kernel hardness index varying from 1.4 to 102.7 was observed in overall collection of 1787 accessions. The high phenotypic variation in kernel hardness index is a reflection of the diverse nature of the scanned germplasm consisting of Chinese cultivars and landraces from 9 geographical regions and 141 foreign accessions from USA, Australia, Europe, and Japan. The distribution of hard and soft type wheats across nine geographical regions broadly divided China into two categories as North and South China. The first category included regions I, II, VIII, and X, where hard type accessions had higher frequency than soft type accession, and the second category consisted of regions III, IV, VI and IX, having more soft type accessions. This pattern of distribution of hard and soft type wheat accessions reflected the breeding preference. The hard type accessions are suitable for wheat noodles and steamed bread, which are preferential daily staple food for people living in North China. Conversely, people in South China prefer rice as staple food and use wheat as secondary food usually for making bakery products like biscuits and cookies etc. The similar distribution of hard and soft type accessions across North and South China was reported previously [10].

In total, six *Pina* and nine *Pinb* variants were detected in 1787 accessions, *Pina-D1a* and *Pinb-D1b* were the most abundant genotypes at *Pina* and *Pinb*, respectively (Table4). Corresponding to this, of 15 *Pina* and *Pinb* combinations, the most extensive distributed combinations were *Pina-D1a*/*Pinb-D1b* (43.1%), followed by *Pina-D1a*/*Pinb-D1a* (32.9%), *Pina-D1a*/*Pinb-D1p* (12.5%) and *Pina-D1b*/*Pinb-D1a* (6.7%) with corresponding average kernel hardness index of 67.7, 31.0, 68.0 and 79.3 (Table 5), which suggest that the order of kernel hardness index between these four combinations from high to low were *Pina-D1b*/*Pinb-D1a*, *Pina-D1a*/*Pinb-D1p*, *Pina-D1a*/*Pinb-D1b* and *Pina-D1a*/*Pinb-D1a* [24].

Regarding to the effects of these allelic variations on kernel hardness, the average kernel hardness index of each combinations are 60.0 or more, except *Pina-D1a*/*Pinb-D1a*, which supporting that wild type genotype (*Pina-D1a*/*Pinb-D1a*) was necessary for showing the soft kernel texture [9, 24]. In addition, the combination *Pina-D1x/Pinb-D1ah* had the highest average kernel hardness than all the remaining genotypes, indicating the null mutations of *Pina* or *Pinb* containing higher kernel hardness than others’ mutations [28].

The wild type *Pina-D1a* was detected as the most frequent allele of *Pina* with frequencies of 91.9, 80.4 and 82.3% in Chinese cultivars, landraces and foreign accessions, respectively (Table 5). Chinese cultivars are prone to have this wild type allele, which could be concluded from previous reports. In Chinese micro-core collection mainly consisting of landraces, *Pina-D1a* had a relatively low frequency (83.6%) [10], while this allele was found to be completely dominant (95.0%) in wheat cultivars released recently from the Yellow and Huai valley of China [11]. The phenomena suggested that this wild type *Pina-D1a* might preserve beneficial agronomic effects selected by breeders. The wild type of *Pinb* (*Pinb-D1a*) and its two mutants (*Pinb-D1b* and *Pinb-D1p*) were frequently detected in Chinese wheat germplasm [10, 11, 15, 19], and the frequency of mutated *Pinb* alleles was much higher than that of mutated *Pina* alleles, supporting the notion that hard wheats in China were mostly due to *Pinb* mutations rather than the ones arising from *Pina* [10, 15, 19]. Moreover, comparing to Chinese landraces and foreign accessions [10, 27], recently released varieties in this work (46.9%) and the Yellow and Huai valley (50.9%) [11] had a relatively high frequency in *Pinb-D1b*, which played a vital role on the improvement of kernel hardness in Chinese wheat breeding. These results are different from the gene pool investigations in North America, Europe, India, CIMMYT and Australia, where both *Pina-D1b* and *Pinb-D1b* alleles are the main sources of kernel hardness [13, 24, 29, 30].

Interestingly, the allele *Pinb-D1c* was completely missing in Chinese cultivars and landraces and was detected only in two foreign accessions. The reports of *Pinb-D1c* in genotypes from Ukraine, Portugal, Finland and European countries [24, 27, 29, 31] supported our finding. The allele *Pinb-D1p* was reported as restricted to Chinese wheat germplasm [10, 11, 15, 17, 19, 27, 28], but in this work we found nine foreign accessions with *Pinb-D1p* (Table 4). Similarly, though the allele *Pina-D1r* and *Pina-D1s* were found restricted to Chinese accessions in international collections of 803 landraces [24], 493 wheat cultivars [27], and 267 wheat cultivars and advanced lines [11], *Pina-D1r* and *Pina-D1s* were characterized in one and five foreign accessions, respectively. These data provided a broad representativeness of wheat accessions collected in this work. The stratified distribution of *Pina* and *Pinb* variants in different geographical regions might have been influenced by repeated use of the core germplasm in breeding and consumer’s preference in a particular region [31].

The simultaneous deletions at both *Pina* and *Pinb* loci are rarely reported so far [27, 28]. In 2005, Ikeda et al. reported the first double null mutation with the presence of D genome and absolute absence of *Pina* and *Pinb* proteins [28]. Later, three Chinese landraces and one Netherlands cultivar were detected absent of both *Pina-D1* and *Pinb-D1* genes, and this mutation was further deduced by primer walking strategy as an approximate 33-kb fragment deletion containing *Pina* and *Pinb* coding regions when compared with the BAC sequence of Chinese Spring on chromosome 5DS [27]. In this work, seven Chinese cultivars and three foreign accessions, designated as *Pina-D1x*/*Pinb-D1ah*, were observed lacking both *Pina-D1* and *Pinb-D1* genes, and these accessions were divided into three groups based on the presence or absence of amplicons surrounding the *Ha* locus on chromosome 5DS, which resulted in three deletion sizes of at least 25-kb, 63-kb and 90-kb, respectively. The accessions with *Pina-D1x*/*Pinb-D1ah* have the highest SKCS index among all 15 genotypes, and these grain textures similar with durum wheat were further observed high degree of adhesion between starch granules and protein matrix through SEM. However, these double null mutants are different from previously characterized double null mutants for the deletion size and kernel hardness [27]. Therefore, these double null mutants could be incorporated into quality improvement in bread wheat though further analysis is needed to clarify their exact deletion size.

## Conclusion

The study herein verified many previous results regarding higher allelic diversity at *Pinb* locus; predominant prevalence of *Pina-D1a* and *Pinb-D1b* alleles; and association of various mutations at *Pina* and *Pinb* loci with SKCS index. The regions I, II, VIII, X foreign accessions we﻿re dominantly represented with hard type wheat while the soft type accessions were more frequent in regions III, IV, VI and IX. The Nei’s diversity, allelic and genotype frequency results together show the promising use of Chinese landraces and foreign accessions to broaden the genetic base of modern Chinese cultivars. The wild type *Pina-D1a* and *Pinb-D1a*, and one *Pinb* mutant (*Pinb-D1b*) are the most common alleles of six *Pina* and nine *Pinb* alleles, and a new double null genotype (*Pina-D1x*/*Pinb-D1ah*) possessed relatively high SKCS hardness index. More hard type varieties were released in recent years with different prevalence of genotypes in different regions. This work would benefit the understanding of the selection and molecular processes of kernel hardness across China and different breeding stages, and provide useful information for the improvement of wheat quality in China.

## Methods

### Wheat germplasm

A total of 1787 accessions were used for this study, including 1539 Chinese cultivars, 107 landraces and 141 accessions originated from USA, Australia, Europe and Japan and grouped together as foreign accessions (Table 1). This panel was obtained from the Institute of Genetics and Developmental Biology, Chinese Academy of Sciences (IGDB, CAS), and the information about the origin and nature of each accession was collected from Chinese Crop Germplasm Resources Information System (http://www.cgris.net/cgris_english.html). All the accessions were grown at the experimental station of the Institute of Genetics and Developmental Biology, Chinese Academy of Sciences, Beijing, in the 2009–2010 cropping season according to local crop management practices. Most accessions are from the winter wheat regions, but a few accessions are from the spring or winter-spring wheat regions, and to secure the survival of all the accessions, the seedlings were covered with a plastic film during the winter season. After harvesting, 300 physiologically mature seeds for each accession were subjected to measuring the kernel hardness using the Perten’s Single Kernel Characterization System (SKCS) 4100. In the successive 2010–2011 growing season, a subset of 623 accessions was grown and harvested for the kernel hardness measurement to assess the effect of years on kernel hardness. Each accession was planted on a 2-m row with inter row spacing of 0.25 cm and plant-to-plant distance of 5 cm within a row.

### DNA isolation

Genomic DNA was extracted from single seedlings of each accessions growing in the greenhouse using CTAB procedure [32]. The isolated DNA was measured with the Nanodrop spectrophotometer (Thermo Fisher Scientific Inc., Wilmington, DE) and diluted to 100 ng/μl for further PCR analysis.

### The modified EcoTILLING

High throughput EcoTILLING analysis was applied to identify *Pina* and *Pinb* alleles in the selected germplasm. The method was followed with Li et al. (2013) with minor modifications based on gene cloning to minimize the use of control DNA templates. Firstly, three dominant allelic variants of *Pina* (*Pina-D1a* from Chinese Spring) and *Pinb* (*Pinb-D1a* from Chinese Spring and *Pinb-D1b* from Neimai 11) were cloned into the pGEM-T Easy vector (Transgen, Beijing, China) as the control samples, respectively. The nested PCR was used to detect *Pina* and *Pinb* allelic variants (Fig. 1; Additional file 1: Table S3). The full length of *Pina* and *Pinb* were amplified individually from genomic DNA of tested samples with primers (Pina-Out-F/R, Pinb-Out-F/R) in the first step PCR reaction. Then, the PCR products and the plasmids (containing *Pina-D1a*, *Pinb-D1a* and *Pinb-D1b*, respectively) were diluted for 100-fold as templates for the second step PCR reaction individually. To improve the amplification efficiencies, the fluorescently labeled primers (LI-COR Biosciences, Lincoln, USA) were mixed with unlabeled primers (Pina-In-F/R, Pinb-In-F/R) in 1:1 ratio for the second step PCR reaction.

### Screening the single nucleotide polymorphism of *Pina* and *Pinb*

For identifying the null alleles of *Pina* and *Pinb*, all of the products of nested PCR were detected with 1% agarose gels electrophoresis. Specific primers were further applied to identify the known null alleles, *Pina-D1b* [20], *Pina-D1r* and *Pina-D1s* [12] (Additional file 1: Table S3). For those samples whose nested PCR products of *Pina* and *Pinb* contained the target bands, different strategies were attempted to identify *Pina* and *Pinb* alleles [10]. The nested PCR products of four tested samples and one control were mixed at the same volumes, and the mixture were denatured and re-annealed to allow the formation of hetero-duplex between the wild type and mutant DNA molecules in a thermocycler as follows: 99 °C for 10 min, 70 cycles of 70 °C for 20 s decreasing 0.3 °C per cycle [33]. The resulted hetero-duplex mixtures were digested with CEL1 enzyme [34], and the cleavage reaction was stopped by adding 5 μl stop solution (2.5 μl 0.25 M EDTA and 2.5 μl formamide loading dye) [10]. To visualize the polymorphisms between the tested samples and the control, 1 μl of CEL1 enzyme digestion product was loaded into 6.5% polyacrylamide gels, and separated on the LI-COR 4300 DNA analyzer (LI-COR Biosciences, Lincoln, USA) at 1500 V/40 watts/45 °C for 4 h. The new allelic variants of *Pina* and *Pinb* were further confirmed by sequencing. The genetic diversity at each locus was calculated using Nei’s index [35] with formula H = 1–∑*Pi* ^*2*^, where H and Pi denote the genetic variation index and the frequency of the number of alleles at the locus, respectively.

For *Pina* and *Pinb* double null mutants, the BAC sequences (CT009735) flanking the *Pina* and *Pinb* genes were download from NCBI to design genome-specific primers surrounding these two genes. Four pairs of specific primers which located on different position of *Pina* and *Pinb* coding sequences were explored to investigate the deletion of *Pina* and *Pinb* in the wheat genomes (Additional file 1: Table S3), which could largely detect the *Pina* and *Pinb* genes in spite of chromosomal rearrangement. Moreover, Nine pairs of primer sets spanning an approximately 90-kb region were designed between −21,803 bp (reference to the ATG of the *Pina* gene) and +68,481 to clarify the molecular mechanism of the *Pina* and *Pinb* double null mutants (Additional file 1: Table S3). The size and position of deletions in these double null mutants were deduced based on the PCR amplification.

### Kernel hardness measurement

The harvested seeds of 1787 accessions were cleaned and kept at dry indoor ventilation for 3 days to bring the moisture content to 11–13%. For each sample, kernel hardness index was measured with 300 seeds through the Perten’s Single Kernel Characterization System (SKCS) 4100 according to the manufacturer’s procedure (Perten Instruments North America Inc., Springfield, IL, USA). Chinese Spring was included as the reference for soft wheat with SKCS index of 25.0 ± 17.0. Regarding to kernel hardness based classification of the germplasm [24, 36], the categories normally include <40.0 (soft), 40.0–60.0 (medium), and >60.0 (hard) though different classification systems have been adopted in different countries [10, 24]. According to these categories, 1787 accessions with different SKCS index were classified into soft (<40.0), medium (40.0–60.0) and hard wheat (>60.0).

### Assessment of grain texture by scanning electron microscopy (SEM)

The SEM images of physiologically mature grains from wheat accessions with double null mutants at *Pina* and *Pinb* loci were compared with those of wild type alleles at *Pina* and *Pinb* loci (*Pina-D1a*/*Pinb-D1a*) and of durum wheat lacking *Pina* and *Pinb* loci. Two grains from each accession were transversely sliced and placed onto glass microscope slides. The slides were fixed with double sided tape, and coated with gold in Dynavac CS300 coating unit [37]. Photographic images were captured at 2000-fold magnification using a ZEISS supra 10 vp field emission scanning electron microscope (Carl Zeiss Microscopy, NY, USA) at 10 kV.

## Additional files

Additional file 1: Table S1. Year effect on SKCS value in 623 accessions. **Table S2.** Number of hard, soft and medium wheat in wheat cultivation regions when regarding to accession type. **Table S3.** Sequence, product size and annealing temperature of PCR primers used for *Pina* and *Pinb* amplification. **Table S4.** SKCS hardness index of *Pina-D1x*/*Pinb-D1ah* genotype. (DOCX 29 kb)

Additional file 2: Figure S1. Identification of *Pina* and *Pinb* deletions in some accessions by a set of selected markers. (A) Primers AGPS-1 was used to check all of DNA quality. (B) Primers Pina-part amplified part of *Pina* coding sequence. (C) Primers Pina-cds amplified the *Pina* coding sequence. (D) Primers Pinb-part amplified part of *Pinb* coding sequence. (E) Primers Pinb-cds amplified the *Pinb* coding sequence. (F) Primers Pina-4 was used to check the deletion of *Pina* and *Pinb* downstream sequence. 1–7 show accessions Chinese Spring, NIL-Novos 67, Yunfengzao 21, Shan 150, 91G 149/Chang 128,865, Hedong TX-008, Xinong 8925–13 respectively. (TIFF 6824 kb)

Additional file 3: Figure S2. Wheat seed protein analysis. (A) Western blot analysis of PINB protein. (B) SDS-PAGE gel of total proteins from wheat mature seeds. Lanes were loaded with 20 μg protein, 1–7 show accessions Chinese Spring, NIL-Novos 67, Yunfengzao 21, Shan 150, 91G 149/Chang 128,865, Hedong TX-008, Xinong 8925–13, respectively. (TIFF 7059 kb)

Additional file 4: Figure S3. Sequence alignments of *Pina* (A) and *Pinb* (B) alleles. (A) *Pina-D1a* (DQ363911), *Pina-D1l* ([6, 15]), and *Pina-D1y*. (B) *Pinb-D1u* (EF620911), *Pinb-D1a* (DQ363913), *Pinb-D1b* (DQ363914), *Pinb-D1c* (KC585019), *Pinb-D1d* (KR259645), *Pinb-D1p* (AY581889), and *Pinb-D1q* (EF620909). (TIFF 7417 kb)

## Abbreviations

ANOVA: Analysis of variance
EcoTILLING: Ecotype targeting induced local lesions in genomes
*H*: Nei’s index of allelic diversity index
NCBI: National Center for Biotechnology Information
nt: Nucleotide
PINA: Puroindoline a
PINB: Puroindoline b
SEM: Scanning electron microscopy
SKCS: Single kernel characterization system
STS: Sequence tagged site
WT: Wild type
