# Supplementary material for: Diversity, distribution of Puroindoline genes and their effect on kernel hardness in a diverse panel of Chinese wheat germplasm
Source: BMC Plant Biol. 2017 Sep 20;17:158. doi: 10.1186/s12870-017-1101-8 (PMC5607584; doi:10.1186/s12870-017-1101-8)
Supplement: Supplementary file 1 — Year effect on SKCS value in 623 accessions. Table S2. Number of hard, soft and medium wheat in wheat cultivation regions when regarding to accession type. Table S3. Sequence, product size and annealing temperature of PCR primers used for Pina and Pinb amplification. Table S4. SKCS hardness index of Pina-D1x/Pinb-D1ah genotype. (DOCX 29 kb) [file 12870_2017_1101_MOESM1_ESM.docx]

**Table S1 Year’s effect on SKCS value in 623 accessions (*P*=0.39)**

| Year | Mean | SE | Minimum | | Maximum | |
| --- | --- | --- | --- | --- | --- | --- |
| 2009-2010 | 49.2 | 23.2 | 1.4 | 97.8 | |  |
| 2010-2011 | 48.0 | 23.4 | 1.1 | 98.0 | |  |

**Table S2 Number of hard, soft and medium wheat in wheat cultivation regions when regarding to accession type**

| Region | Accession Type | Soft wheat | Hard wheat | Medium wheat | |
| --- | --- | --- | --- | --- | --- |
| I | Cultivar | 26 | 188 | 30 |  |
|  | Landrace | 0 | 3 | 1 |  |
| II | Cultivar | 294 | 652 | 119 |  |
|  | Landrace | 7 | 17 | 1 |  |
| III | Cultivar | 39 | 35 | 7 |  |
|  | Landrace | 16 | 8 | 1 |  |
| IV | Cultivar | 58 | 32 | 5 |  |
|  | Landrace | 19 | 17 | 7 |  |
| V | Cultivar | 1 | 1 | 0 |  |
|  | Landrace | 1 | 0 | 0 |  |
| VI | Cultivar | 5 | 4 | 0 |  |
|  | Landrace | 3 | 0 | 0 |  |
| VIII | Cultivar | 10 | 20 | 2 |  |
|  | Landrace | 1 | 3 | 0 |  |
| IX | Cultivar | 1 | 0 | 0 |  |
|  | Landrace | 2 | 0 | 0 |  |
| X | Cultivar | 3 | 6 | 1 |  |
|  | Landrace | 0 | 0 | 0 |  |

**Table S3 Sequence, product size and annealing temperature of PCR primers used for *Pina* and *Pinb* amplification**

| Primer name | Forward primer | Reverse primer | Tm^¶^ | Fragment size (bp) |
| --- | --- | --- | --- | --- |
| Pina-Out | TGTGCCGAATCTCAATCT | TTGGTGCATATCATTCTTG | 54 | 1933 |
| Pina-In | CAGGAAGCGACATGTATCTCAAT | AATGGTATCCTCACGGCAAACTCA | 63 | 1239 |
| Pinb-Out | TTATCGCAACATCCGGAAAGT | AAGTTGTTGGATGGACGAATAAGGTT | 62 | 1877 |
| Pinb-In | CCAACGAAACTAATGAGAAATAAAAAGGTG | AAGTTGTTGGATGGACGAATAAGGTT | 63 | 1421 |
| Pina-D1b | AATACCACATGGTTCTAGATACTG | GCAATACAAAGGACCTCTAGATT | 60 | 776 |
| Pina-D1r | TTGGATTTCCGCAGCTAGAT | CATCCACACGCCTCTGTTCT | 58 | 2700 |
| Pina-D1s | TTCAAAAGTTTGCGGGGACC | CATCCACACGCCTCTGTTCT | 58 | 1600 |
| Pina-1 | CCTTACCTCGTTCATCCTTA | GATGTCGTCCTCTTCTACCC | 53 | 784 |
| Pina-2 | CAGGAAGCGACATGTATCTCAAT | AATGGTATCCTCACGGCAAACTCA | 65 | 1239 |
| Pinb-3 | CCAACGAAACTAATGAGAAATAAAAAGGTG | AAGTTGTTGGATGGACGAATAAGGTT | 64 | 1421 |
| Pina-4 | GCCACAAGACACGCATGACG | CGACTGAGCAGGATAGGAGGAA | 61 | 508 |
| Pina-5 | GAGCCTCAAACTGGAAGCACA | ACGACAGCGGCGAGTATGGA | 64 | 350 |
| Pina-6 | CATTTGCCACTGCCTTTAGC | AAGTCGTCGGTGACCTCCTC | 59 | 630 |
| Pina-7 | GCCCAAGTGATGAGGTAGAGG | CGAATCCCAGGAGAAAGGTG | 59 | 593 |
| Pina-8 | CAAGGACAAAGGGCAGATAGG | AGCAATGGTGGAGAAGTGGG | 60 | 491 |
| Pina-9 | TTTGGTGTAGGTAAACCGAATG | GACAAGGACTCCGACGATGA | 58 | 872 |
| Pina-part | CTGCTTGCTCTGGTAGCGAG | CAGGTTCTTGGCTTCTTGTATCAC | 58 | 360 |
| Pinb-part | ATGAAGACCTTATTCCTCCTAGCTCTC | ATTGTGGTGCTATCTGGCTCA | 58 | 286 |
| Pina-cds | CATCTATTCATCTCCACCTGC | GTGACAGTTTATTAGCTAGTC | 58 | 524 |
| Pinb-cds | GAGCCTCAACCCATCTATTCATC | CAAGGGTGATTTTATTCATAG | 58 | 597 |
| AGPS-1 | CAGGGTACTGCAGATGCTGTA | CGGAGAAGCTGAAGCATCA | 56 | 636 |

^¶^ Tm=PCR annealing temperature (^o^C).

**Table S4 SKCS hardness index of *Pina-D1x /Pinb-D1ah* genotype and the PCR amplification with primers surrounding the *Pina* and *Pinb* genes**

| Primer name | Forward primer ^a^ | Reverse primer ^a^ | NIL-Novos 67 | Yunfengzao 21 | 06-01216 | Kelao 4 | Shan 150 | Victory | 91G 149/  Chang 128865 | Hedong TX-008 | Xinong 8925-13 | Vendvr | XY81 | NX4184 |
| --- | --- | --- | --- | --- | --- | --- | --- | --- | --- | --- | --- | --- | --- | --- |
| Pina-1 | -21803 | -21019 | ﹢^b^ | ﹢ | ﹢ | ﹢ | ﹢ | N^c^ | N | N | N | N | ﹢ | N |
| Pina-2 | -435 | 804 | N | N | N | N | N | N | N | N | N | N | ﹢ | N |
| Pinb-3 | 17584 | 19005 | N | N | N | N | N | N | N | N | N | N | ﹢ | N |
| Pina-4 | 23862 | 24370 | N | N | N | N | N | N | N | N | N | N | ﹢ | N |
| Pina-5 | 24242 | 24592 | N | N | N | N | N | N | N | N | N | N | ﹢ | N |
| Pina-6 | 41214 | 41844 | ﹢ | ﹢ | ﹢ | ﹢ | ﹢ | N | N | N | N | N | ﹢ | N |
| Pina-7 | 64047 | 64640 | ﹢ | ﹢ | ﹢ | ﹢ | ﹢ | ﹢ | N | N | N | N | ﹢ | N |
| Pina-8 | 66691 | 67182 | ﹢ | ﹢ | ﹢ | ﹢ | ﹢ | ﹢ | N | N | N | N | ﹢ | N |
| Pina-9 | 67609 | 68481 | ﹢ | ﹢ | ﹢ | ﹢ | ﹢ | ﹢ | N | N | N | N | ﹢ | N |
| SKCS index | | | 96.9 ± 16.5 | 95.6 ± 13.2 | 92.4 ± 16.3 | 97.8 ± 14.5 | 88.8 ± 15.0 | 77.1 ± 13.8 | 89.0 ±15.4 | 92.3 ± 12.4 | 90.7 ± 14.4 | 90.2 ± 15.3 | 75.7 ± 12.5 | 88.1 ± 15.4 |

^a^ Position (Reference to ATG of Pina); ^b^ and ^c^ indicate success (﹢) and failure (N) of expected PCR amplicons.
