# Supplementary figures and images for: Diversity, distribution of Puroindoline genes and their effect on kernel hardness in a diverse panel of Chinese wheat germplasm
Source: BMC Plant Biol. 2017 Sep 20;17:158. doi: 10.1186/s12870-017-1101-8 (PMC5607584; doi:10.1186/s12870-017-1101-8)

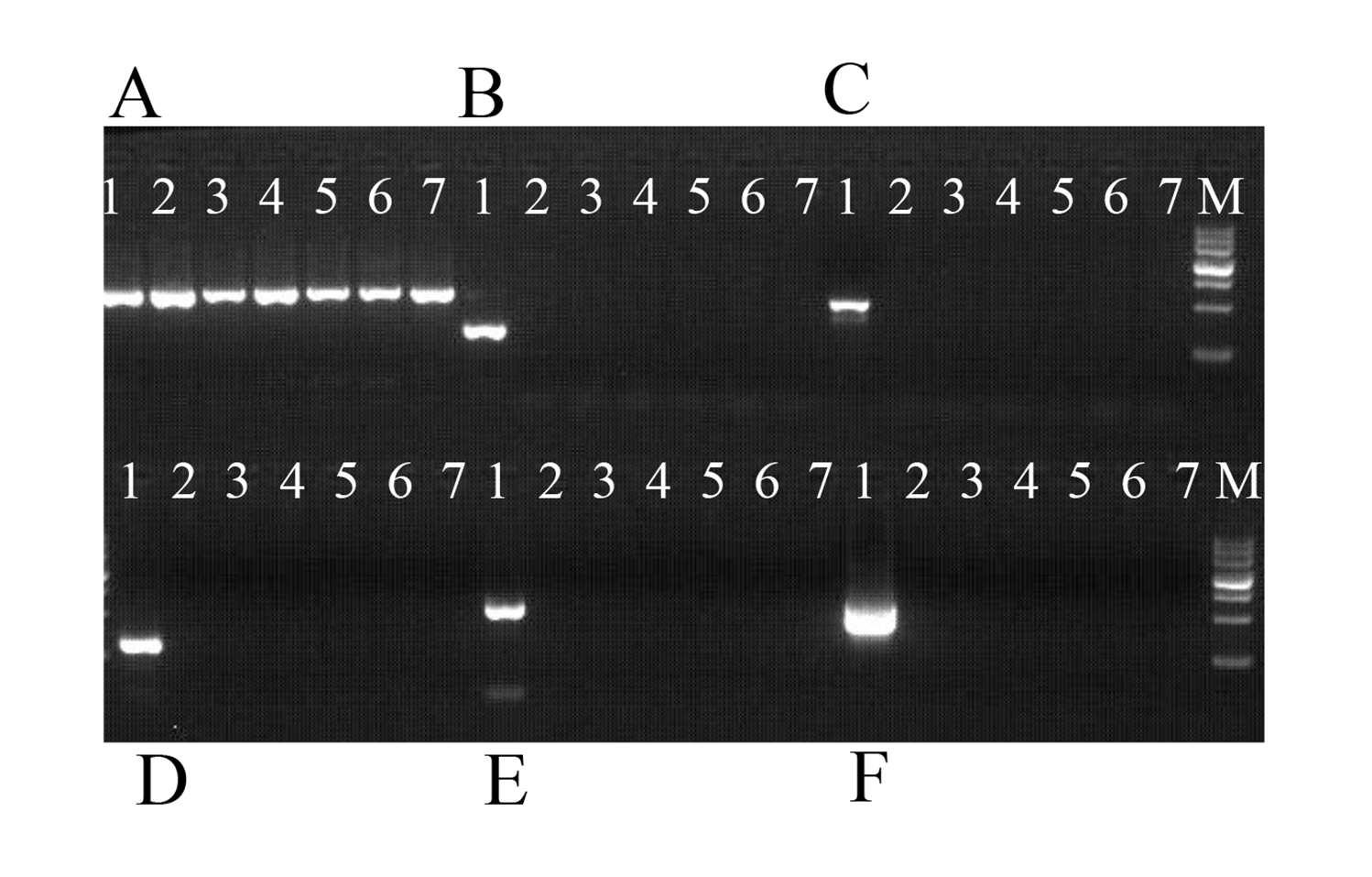

Supplement: Supplementary file 2 — Identification of Pina and Pinb deletions in some accessions by a set of selected markers. (A) Primers AGPS-1 was used to check all of DNA quality. (B) Primers Pina-part amplified part of Pina coding sequence. (C) Primers Pina-cds amplified the Pina coding sequence. (D) Primers Pinb-part amplified part of Pinb coding sequence. (E) Primers Pinb-cds amplified the Pinb coding sequence. (F) Primers Pina-4 was used to check the deletion of Pina and Pinb downstream sequence. 1–7 show accessions Chinese Spring, NIL-Novos 67, Yunfengzao 21, Shan 150, 91G 149/Chang 128,865, Hedong TX-008, Xinong 8925–13 respectively. (TIFF 6824 kb) [file 12870_2017_1101_MOESM2_ESM.tif]

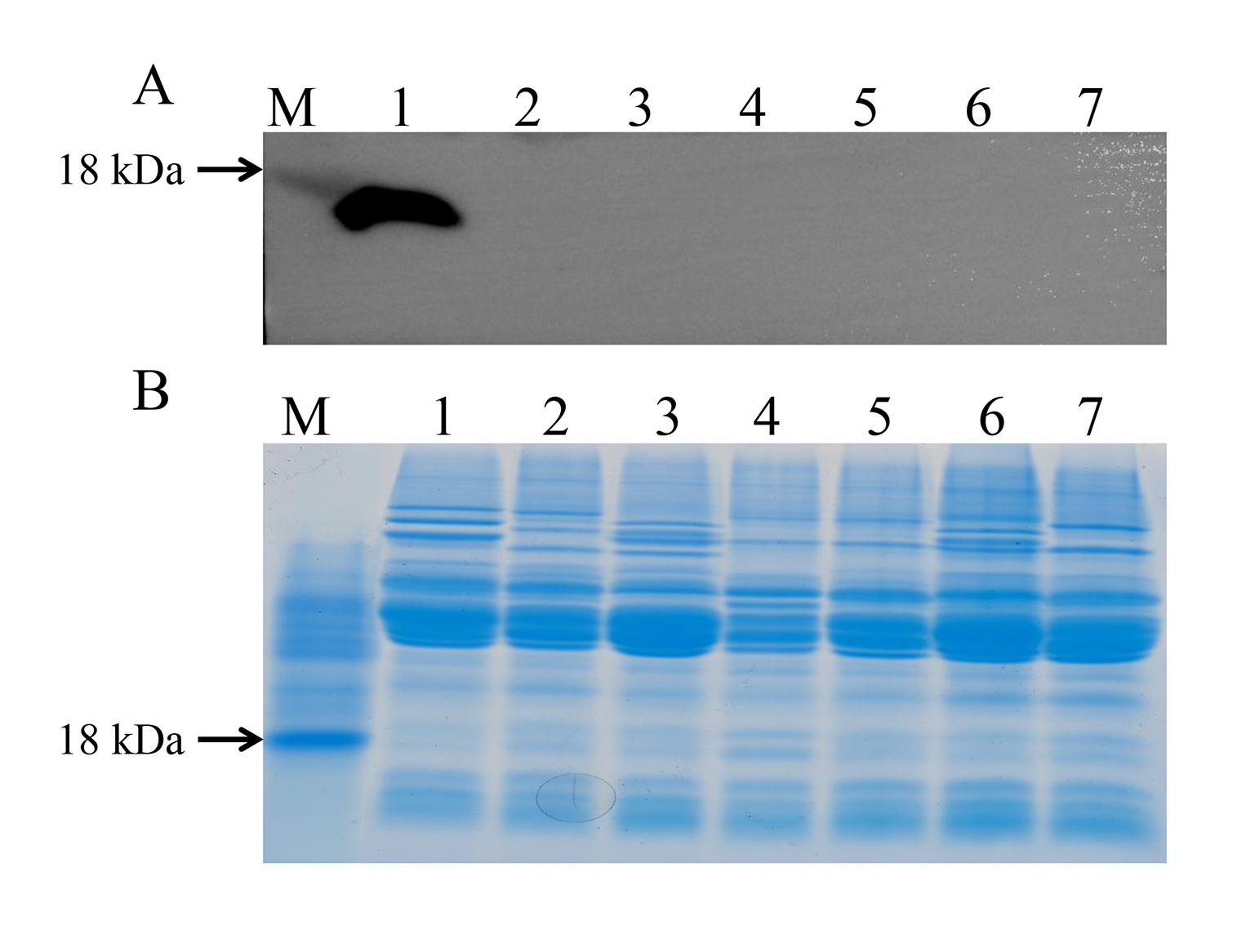

Supplement: Supplementary file 3 — Wheat seed protein analysis. (A) Western blot analysis of PINB protein. (B) SDS-PAGE gel of total proteins from wheat mature seeds. Lanes were loaded with 20 μg protein, 1–7 show accessions Chinese Spring, NIL-Novos 67, Yunfengzao 21, Shan 150, 91G 149/Chang 128,865, Hedong TX-008, Xinong 8925–13, respectively. (TIFF 7059 kb) [file 12870_2017_1101_MOESM3_ESM.tif]

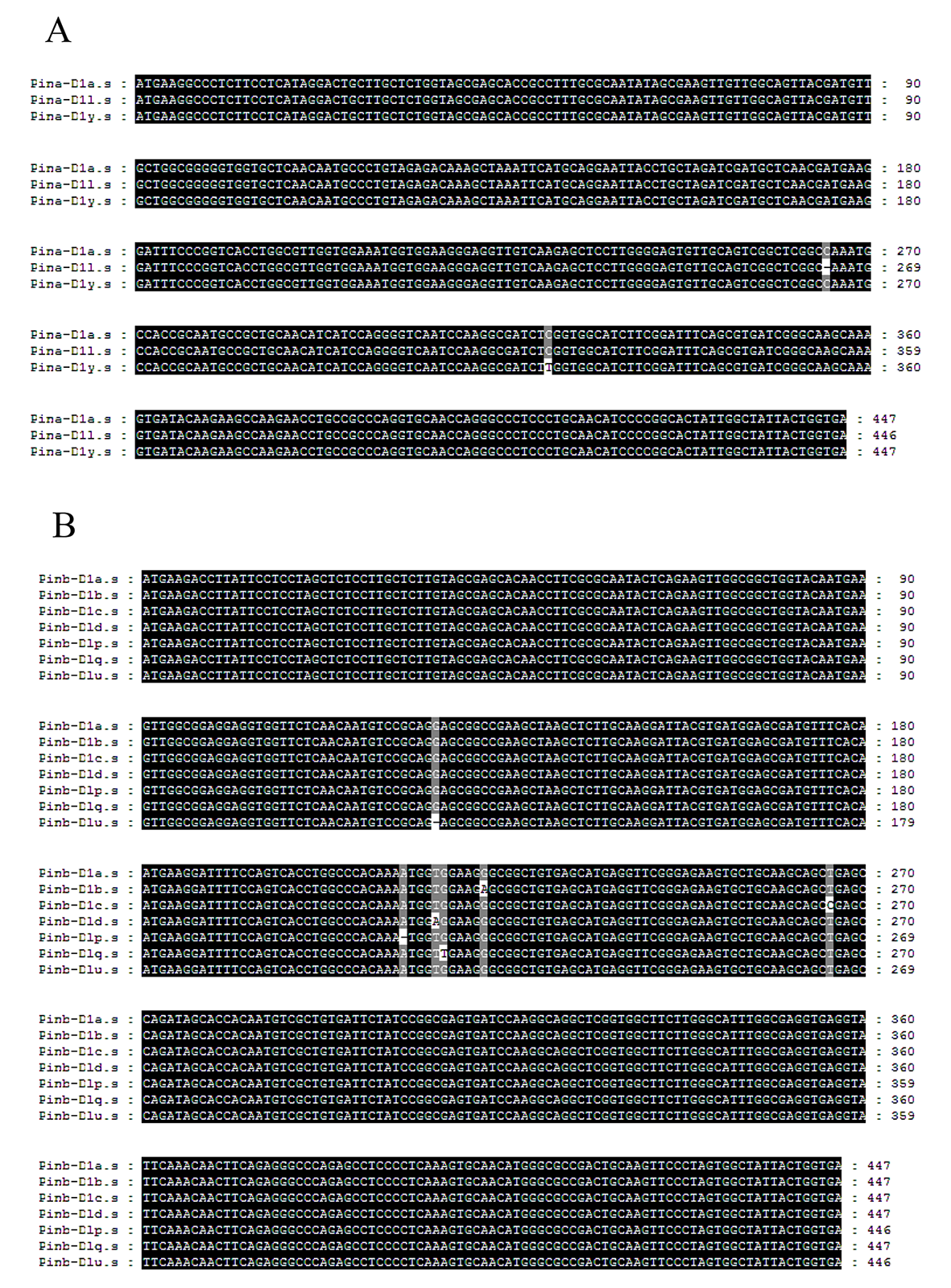

Supplement: Supplementary file 4 — Sequence alignments of Pina (A) and Pinb (B) alleles. (A) Pina-D1a (DQ363911), Pina-D1l ([6, 15]), and Pina-D1y. (B) Pinb-D1u (EF620911), Pinb-D1a (DQ363913), Pinb-D1b (DQ363914), Pinb-D1c (KC585019), Pinb-D1d (KR259645), Pinb-D1p (AY581889), and Pinb-D1q (EF620909). (TIFF 7417 kb) [file 12870_2017_1101_MOESM4_ESM.tif]
